# Pressure distributions inside intervertebral discs under unilateral pedicle screw fixation in a porcine spine model

**DOI:** 10.1186/s13018-018-0962-3

**Published:** 2018-10-16

**Authors:** Zhao Meng, Chen Wang, Li-Jun Tian, Xue-Jun Zhang, Dong Guo, Yan Zou

**Affiliations:** 1 Department of Orthopaedics, Children’s Hospital of Hebei Province, No.133, Jianhua Street, Yuhua District, Shijiazhuang, 050031 China; 2 Department of Orthopaedics, the Third Hospital of Shijiazhuang, No. 15 South of Tiyu Street, Shijiazhuang, 050011 Hebei China; 3 Department of Orthopaedics, Beijing Children’s Hospital, Capital Medical University, No. 56 Nan-li-shi Road, Beijing, 100045 China

**Keywords:** Intervertebral disc, Pressure, Pedicle screw fixation, Porcine, Spine, Unilateral

## Abstract

**Background:**

Little data are available regarding the effects of pedicle screws on the intervertebral disc stress for different spinal segments. The aim of this study was to analyze the intervertebral disc stress in response to the placement of pedicle screws.

**Methods:**

T3–4, T11–12, T15–L1, L3–4, and L4–5 intervertebral disc segments from six porcine spine specimens were harvested. A compressive load of 200 N was applied both before and after the pedicle screw was implanted on the left side of each target segment; the resulting pressure was measured during vertical, 5° anterior flexion, 5° posterior extension, and 5° lateral bending.

**Results:**

The posterior intradiscal pressures of the intervertebral disc were significantly lower in the fixed group than in the unfixed group for all segments during vertical, 5° anterior flexion, and 5° posterior extension. The left pressures of the intervertebral disc were significantly lower in the fixation group for all segments. During 5° lateral bending, the left intervertebral disc pressures were significantly lower in the fixation group. Lower mean pressures were observed in the fixed group.

**Conclusions:**

Unilateral pedicle screws can effectively reduce the pressure of the fixed lateral intervertebral disc. Moreover, it can change the pressure distribution of the intervertebral disc and reduce the pressure of the entire intervertebral disc, especially the posterior side of the intervertebral disc.

## Background

Scoliosis, one of the complex three-dimensional deformities of the spine, refers to a lateral curvature of the spine in the coronal plane of more than 10°. It has a high prevalence of about 1/1000 and is usually complicated with the spine rotation and change of numbers of the sagittal dorsal or anterior processes, as well as uneven rib levels, the pelvic rotation and tilt, and paraspinal ligament and muscle abnormalities [1, 2]. Scoliosis is often used generically to refer to all spinal deformities in children. It can be categorized into three major types, congenital, syndromic, and idiopathic [3, 4]. Progressive scoliosis will seriously affect the children’s skeletal growth and the development of various organs, and severe idiopathic scoliosis and most of congenital scoliosis require surgery.

Growing rods have been the mainstay surgical treatment of scoliosis. As early as 1963, Harrington first advocated the use of non-fusion method of internal fixation surgery for scoliosis [5]. Similarly, in 2001, Blakemore et al. reported a new generation of non-fusion internal fixation; although only a preliminary report, a significant improvement of the Cobb angle was observed post-operation [6]. Recently, pedicle screw-rod constructs have become increasingly popular in the treatment of spinal deformities, as they have excellent biomechanical properties and are suitable for the transfer and subsequent maintenance of large correction forces in all planes [7].

However, most biomechanical studies of scoliosis focus on pathology and morphology, and regarding the scoliosis internal fixation system, most studies focus on the clinical efficacy. To our knowledge, there are little available data regarding the effects of pedicle screws on the intervertebral disc stress for different spinal segments. At present, the clinical application of unilateral pedicle screw fixation for the treatment of scoliosis in children has been established; thus, the purpose of this study was to explore the feasibility of unilateral pedicle screw fixation for scoliosis based on bilateral pedicle screw fixation, with less interference and fewer implants to achieve the same control and orthopedic purposes. However, there is a lack of biomechanical basis and little data are available regarding the effects of unilateral pedicle screws on the intervertebral disc stress for different spinal segments. Most of the studies used sensors and probes in order to evaluate the pressure data of the intervertebral disc, and a shortcoming is that the results are only from certain points and also not intuitive. Pressure-sensitive film can intuitively display the pressure characteristics of each segment in the upper thoracic and lumbar spine [8] and thus is more appropriate in investigating the pressure distribution of the intervertebral disc. In this study, this approach was used to observe the pressure conditions of the intervertebral disc. Immature pigs are usually used as animal models to test the spinal internal fixation system for scoliosis modeling, because they have a similar anatomical structure to humans and their growth cycle is suitable for disease progress research. Therefore, we chose the porcine spine as the experimental subjects [9–11]. Accordingly, our study was established to analyze the intervertebral disc stress in response to the placement of unilateral pedicle screws. The data might be helpful for the clinical management of scoliosis.

## Methods

### Segmentation of specimens

Six spine specimens of 6-week-old female pigs were obtained from the Kangning Co., Ltd., Zhuozhou City, China. Spines were examined by CT scan; all six porcine spines showed no deformity, tumor, fracture, and other lesions. The characteristic spinal segments were taken as follows: T3–4, T11–12, T15–L1, L3–4, L4–5 segments and their adjacent superior and inferior vertebral bodies. The study was approved by the ethics committee of the local hospital.

### Biomechanics experiment

**Fig. 1:**
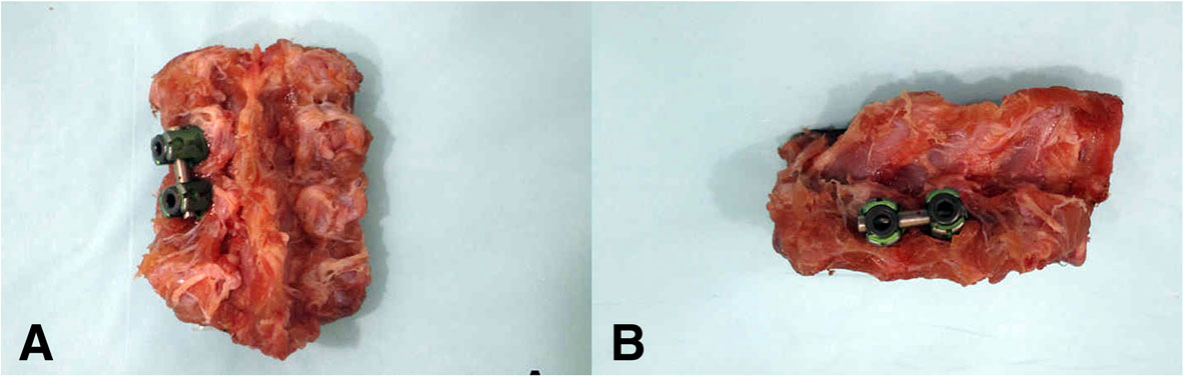
Representative images of the posterior cervical fixation pedicle screw system. **a** Back view of the T11–12 segment. **b** Side view of the T11–12 segment

**Fig. 2:**
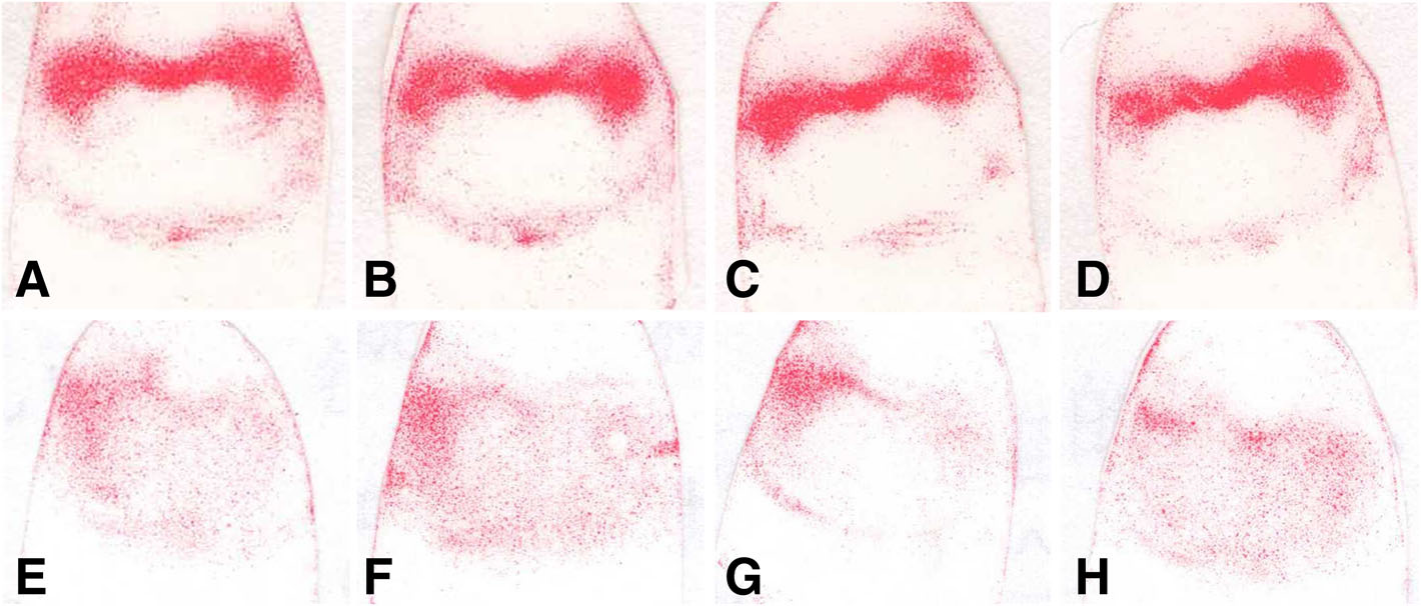
Representative images (T11–L2) of pressure-sensitive film in the unfixed and fixed groups under different loading conditions. **a**–**d** Vertical, 5° anterior flexion, 5° posterior extension, and 5° lateral bending in the unfixed group. **e**–**h** Vertical, 5° anterior flexion, 5° posterior extension, and 5° lateral bending in the fixed group

All specimens were sealed with polyethylene films and stored at − 20 °C. The specimens were equilibrated for about 12 h at 3 °C and wrapped in polyethylene films to maintain the humidity [12]. Then, the sections of the up- and down-ends of specimens were embedded properly with self-condensing dental base acrylic resin powder with a thickness of 1 cm. The experimental environment was maintained at a temperature of 20 °C and a humidity of 44%. After being placed onto the biomechanics experimental machine (Model CSS-44020, Changchun Research Institute for Testing Machines, China), an axial vertical compressive load of 300 N was applied for each segment to reduce the impact of the over-hydration effect of the intervertebral disc [12].The target segment intervertebral disc was carefully cut, without destroying the anterior and posterior longitudinal ligament and articular process. Then, the shape-adjusted and pressure-sensitive film was placed in the intervertebral disc and sealed with plastic membrane. The prepared spinal segments were again fixed in the biomechanical experimental machine, and a vertical compressive load of 200 N was applied; before the loading, three times of the pre-loading were applied in order to eliminate the viscoelasticity. The 2-min method was used for the pressure-sensitive film detection, that is, after development for 5 s, the film was maintained for 2 min to get a more stable and uniform image. A consecutive pressure loading was applied for the same spinal segment, and each pressure was loaded after the replacement of pressure-sensitive film. When the results of the three experiments are similar, the last pressure-sensitive film was analyzed. In order to simulate 5° anterior flexion, posterior extension, and lateral bending, a 5° wedge-shaped bevel was prepared by self-condensing dental base acrylic resin powder (type II, Shanghai Medical Devices Co., Ltd., China) and placed between the experimental machine and the model before loading the compressive force [13]. Thereafter, the posterior cervical fixation pedicle screw system (Shandong Weigao Orthopedic Device Co., Ltd., China) was implanted on one side (left side) of each target segment (Fig. 1); after the placement was confirmed by X-ray examination, the loading procedure was repeated. The appropriate pressure-sensitive film was retained for both the unfixed and fixed groups (for representative images, see Fig. 2).

#### Groups and data analysis

For both of the unfixed and fixed groups, the following pressure data were recorded and analyzed: the left, right, posterior, and mean pressure under vertical loading; the left, right, posterior, and mean pressure under 5° anterior flexion or posterior extension; and the left pressure under 5° lateral bending. Data were extracted from the double-sided pressure-sensitive paper (super low-pressure type: LLW) using pressure prescale spectrophotometer (Fujitsu, Japan); the same data was recorded by the same researcher in 1 mm successively, and then, an appropriate range was selected to calculate the mean pressure. The range of the posterior intervertebral disc is the posterior one third of the vertebral body and the intervertebral disc. The range of the front annulus is the anterior two thirds of the vertebral body and the intervertebral disc. The mean pressure was calculated as the average value of the entire intervertebral disc; the range of the 5° anterior flexion, posterior extension, or lateral bending is separated by the middle line.

### Statistical analysis

The experimental data were expressed as mean ± standard deviation. The data were analyzed by SPSS13.0 statistical software (SPSS Inc., IL, USA). The data of each group were analyzed by Shapiro-Wilk normality test and paired Student’s *t* test. A value of *P* < 0.05 was considered statistically significant.

## Results

### The posterior intradiscal pressures under different loading conditions

When comparing the posterior intradiscal pressures of intervertebral disc (Table 1), in the unfixed group, generally, there was no significant difference among different loading conditions; however, the posterior intradiscal pressure under 5° posterior extension for the T3–4 segment was significantly lower than those under vertical or 5° anterior flexion conditions (*P* < 0.05). And for the comparison between the fixed and unfixed groups, under vertical loading condition, the posterior intradiscal pressures in the fixed group were significantly lower than those in the unfixed group for all spinal segments (except the T3–4 segment) (*P* < 0.05). Under 5° anterior flexion loading condition, the posterior intradiscal pressures in the fixed group were significantly lower than those in the unfixed group for all spinal segments (except the L4–5 segment) (*P* < 0.05). Under 5° posterior extension condition, the posterior intradiscal pressures in the fixed group were significantly lower than in those the unfixed group for all spinal segments (except the T3–4 segment) (*P* < 0.05).

**Table 1:** The posterior intradiscal pressures under different loading conditions in the unfixed and fixed groups (MPa)

Segment	Vertical		5° anterior flexion		5° posterior extension	
	Unfixed	Fixed	Unfixed	Fixed	Unfixed	Fixed
T3–4	1.83 ± 0.23	1.48 ± 0.25	1.95 ± 0.22	1.31 ± 0.16^†^	1.15 ± 0.12*	1.90 ± 0.27
T11–12	2.88 ± 0.27	1.02 ± 0.17^†^	2.66 ± 0.44	0.98 ± 0.18^†^	3.07 ± 0.39	1.11 ± 0.20^†^
T15–L1	2.51 ± 0.47	0.70 ± 0.14^†^	2.39 ± 0.279	0.67 ± 0.15^†^	2.31 ± 0.30	0.87 ± 0.10^†^
L3–4	1.94 ± 0.30	0.88 ± 0.20^†^	1.69 ± 0.33	0.86 ± 0.14^†^	1.87 ± 0.16	1.27 ± 0.23^†^
L4–5	1.87 ± 0.51	1.09 ± 0.20^†^	1.67 ± 0.33	1.15 ± 0.20	1.91 ± 0.36	1.10 ± 0.22^†^

*P < 0.05 versus the corresponding values of the unfixed group under vertical or 5° anterior flexion loading condition

^†^*P* < 0.05 versus the corresponding values of the unfixed group under different loading conditions

### Left and right disc pressures under different loading conditions

The left and right pressures under different loading conditions in the unfixed and fixed groups were shown in Table 2. As for the unfixed group, there were no significant difference regarding the intervertebral disc pressures between the left and right sides for all spinal segments under the loading condition of vertical, 5° anterior flexion, and 5°posterior extension. As for the fixed group, left intervertebral disc had significantly lower pressures for all spinal segments under vertical loading condition (*P* < 0.05), lower pressures for all spinal segments (except the T3–4 segment) under 5° anterior flexion (*P* < 0.05), and lower pressures for all spinal segments (except the T3–4 and T15–L1 segments) under 5° posterior extension (*P* < 0.05), when compared to those of the right side.

**Table 2:** The left and right pressures under different loading conditions in the unfixed and fixed groups (MPa)

Segment	T3–4	T11–12	T15–L1	L3–4	L4–5
Vertical					
Unfixed (L)	1.22 ± 0.25	1.39 ± 0.23	1.44 ± 0.24	1.23 ± 0.18	1.30 ± 0.17
Unfixed (R)	1.30 ± 0.28	1.46 ± 0.18	1.52 ± 0.25	1.32 ± 0.20	1.27 ± 0.21
Fixed (L)	1.05 ± 0.13^a^	0.59 ± 0.10^a,b^	0.45 ± 0.08^a,b^	0.75 ± 0.18^a,b^	0.76 ± 0.13^a,b^
Fixed (R)	1.46 ± 0.35	1.14 ± 0.12	0.81 ± 0.11	1.16 ± 0.16	1.53 ± 0.28
5° anterior flexion					
Unfixed (L)	1.43 ± 0.24	1.68 ± 0.23	1.48 ± 0.30	1.47 ± 0.12	1.12 ± 0.25
Unfixed (R)	1.49 ± 0.32	1.41 ± 0.31	1.53 ± 0.22	1.56 ± 0.32	1.00 ± 0.15
Fixed (L)	1.04 ± 0.14^b^	0.63 ± 0.11^a,b^	0.51 ± 0.07^a,b^	0.60 ± 0.08^a,b^	0.73 ± 0.06^a,b^
Fixed (R)	1.22 ± 0.14	1.31 ± 0.29	0.72 ± 0.07	0.86 ± 0.07	1.36 ± 0.29
5° posterior extension					
Unfixed (L)	0.93 ± 0.03	1.70 ± 0.29	1.41 ± 0.30	0.91 ± 0.13	1.48 ± 0.28
Unfixed (R)	1.01 ± 0.16	1.86 ± 0.22	1.65 ± 0.25	1.02 ± 0.18	1.34 ± 0.24
Fixed (L)	1.26 ± 0.23^b^	0.44 ± 0.07^a, b^	0.65 ± 0.12^b^	0.67 ± 0.14^a,b^	0.68 ± 0.11^a,b^
Fixed (R)	1.39 ± 0.27	1.35 ± 0.20	0.82 ± 0.11	1.32 ± 0.20	1.36 ± 0.23
5° left lateral bending					
Unfixed (L)	1.70 ± 0.18^c,d^	1.79 ± 0.14^c,d^	1.84 ± 0.22^c,d^	1.74 ± 0.28^c,d^	1.60 ± 0.23^c,d^
Fixed (L)	0.83 ± 0.12	0.93 ± 0.12	0.50 ± 0.05	0.60 ± 0.10	0.55 ± 0.08

^a^*P* < 0.05 versus the corresponding right pressures of the fixed group under different loading conditions

^b^*P* < 0.05 versus the corresponding left pressures of the unfixed group under different loading conditions

^c^*P* < 0.05 versus the left pressures of the unfixed group under vertical loading condition

^d^*P* < 0.05 versus the corresponding left pressures of the fixed group under 5° left lateral bending loading condition

In the unfixed group, the left intervertebral disc pressures for all spinal segments under 5° left lateral bending were significantly higher than those under vertical loading condition (*P* < 0.05). The fixed group had significantly lower left intervertebral disc pressures for all spinal segments (except the T3–4 segment) under vertical loading condition (*P* < 0.05), lower values for all spinal segments under 5° anterior flexion (*P* < 0.05), lower values for all spinal segments (except the T3–4 segment) under 5°posterior extension (*P* < 0.05), and lower values for all spinal segments under 5° left lateral bending (*P* < 0.05), when compared to those in the unfixed group.

### Disc pressures of the entire intervertebral disc under different loading conditions

The mean pressures of intervertebral disc were analyzed (Table 3). The fixed group had lower mean pressures in all segments (except the T3–4 segment) under vertical loading condition (*P* < 0.05), lower mean pressures in all segments (except the T3–4 and L4–5 segments) under 5° anterior flexion loading condition (*P* < 0.05), and lower mean pressures in all segments (except the T3–4 and L3–4 segments) under 5° posterior extension loading condition (*P* < 0.05), when compared to the unfixed group.

**Table 3:** The mean pressure under different loading conditions in the unfixed and fixed groups (MPa)

Segments	Vertical			5° anterior flexion			5° posterior extension		
	Unfixed	Fixed	Unfixed/fixed (%)	Unfixed	Fixed	Unfixed/fixed (%)	Unfixed	Fixed	Unfixed/fixed (%)
T3–4	1.29 ± 0.22	1.16 ± 0.24	89.64	1.52 ± 0.19	1.17 ± 0.18	77.03	1.06 ± 0.11	1.23 ± 0.25	115.86
T11–12	1.69 ± 0.34	0.93 ± 0.17*	54.95	1.86 ± 0.33	0.91 ± 0.16*	48.95	1.93 ± 0.20	0.90 ± 0.16*	46.60
T15–L1	1.53 ± 0.32	0.67 ± 0.04*	43.80	1.73 ± 0.28	0.58 ± 0.11*	33.66	1.39 ± 0.25	0.75 ± 0.14*	53.82
L3–4	1.30 ± 0.18	0.93 ± 0.14*	71.24	0.97 ± 0.19	0.86 ± 0.12*	88.57	1.22 ± 0.20	1.09 ± 0.22	89.10
L4–5	1.48 ± 0.26	1.11 ± 0.18*	74.71	1.11 ± 0.21	1.05 ± 0.22	95.21	1.47 ± 0.27	1.06 ± 0.18*	72.10

*P < 0.05 versus the corresponding value of the unfixed group

The percentage of the mean pressure of the fixed group to that of unfixed group was calculated. Higher percentage indicated less effects of the fixation between specific intervertebral discs. The highest percentage was found in the T3–4 segment, and the lowest value was found in the T15–L1 segment under vertical loading condition. The highest percentage was found in the L4–5 segment, and the lowest in the T15–L1 segment under 5° anterior flexion loading condition. The highest percentage was found in the T3–4 segment, and the lowest in the T11–12 segment under 5° posterior extension loading condition.

## Discussion

Previously, scoliosis-related studies mainly focused on the histomorphology, microscopic examination, and radiographic analysis [14–19]. However, limited information is available regarding the specific pressure data of intervertebral disc when an asymmetry stress was present, and most of the studies used sensors and probes in order to evaluate the pressure, and a shortcoming is that the results are only from certain points and also not intuitive [13, 20]. In contrast, the pressure-sensitive film method is more appropriate in investigating the pressure distribution of the intervertebral disc [8]. In the present study, pressure characteristics for different spinal segments under compression were recorded and analyzed using the pressure-sensitive film method. Previously, pigs are widely used for experimental purposes in spinal research and implant testing, mainly because of the anatomical similarities to humans [9–11]; therefore, we chose the porcine spine as the experimental subjects.

At present, there are many methods for the treatment of congenital scoliosis clinically, such as anterior and posterior convex epiphysiodesis, hemivertebra resection, and vertical expandable prosthetic titanium rib [21]. Some of these technologies have good effects, while some have not achieved the desired results; the unsatisfactory curative effect may partially be due to the lack of experimental research on spine biomechanics details or depth. Our study may lay foundation for further research on the biomechanics of the spinal column and provide ideas for the improvement and optimization of clinical therapy techniques. Our study proved that short-segment pedicle screw fixation is a minimally invasive and effective treatment method, which can significantly reduce the pressure on the fixed side of the spine and improve the unbalanced force distribution. This technique can be used for the surgical intervention of short-segment malformations in patients with congenital scoliosis, as well as severe long-spine-affected scoliosis, thereby achieving spinal deformity control with less vertebral body interference and implants, as well as minimizing the impact on children’s spine development.

Regarding the non-fixed status, darker color in the back side of the film, which indicated higher pressure, was observed under vertical, 5° flexion, and 5°posterior extension loading conditions for all the spinal segments. In previous studies, the frontside pressure increased under the flexion, whereas the backside pressure decreased, and vice versa under the posterior extension [13, 22–24]. Notably, in this study, the backside pressure of the T3–4 segment was significantly reduced under posterior extension as compared with under vertical or 5° anterior flexion loading condition, and there was no significant difference in the backside pressure values among the other groups under different loading conditions for the same segment, although an increasing tendency was observed for the T11–12, L4–5 segments when under 5° posterior extension. The special physiological structure of the T3–4 segment may contribute to the effect observed in the present study, as the upper thoracic discs were relatively smaller, with more vertebral posterior column structures, the pressure was partially taken by articular process and therefore the intervertebral disc may take less pressure as expected under posterior extension [12, 25].

The left and right side pressures were basically the same under different loading conditions for all the spinal segments without significant differences. When under the 5° left lateral bending, as expected, the balance of pressure receiving between the two sides was broken; higher pressure was recorded on the concave side compared to the convex side, which is also consistent with a previous report [26].

For the fixed group, asymmetric pressures were observed between the left and right side under vertical, 5° flexion, and 5° posterior extension conditions, and this phenomenon has been reported previously [24]. As expected, the fixed side pressure was less than those of the unfixed side; significant differences were found for most of the spinal segments.

In this study, we obtained the specific pressure value of different spinal segments under asymmetric stress, which is valuable to test the effects of the pedicle screw fixation system on correcting of asymmetric stress. When under 5° left lateral bending condition, the left side pressures were significantly reduced as compared with the unfixed group for segments. It is generally believed that the wedge deformation of the vertebral body and intervertebral disc is essential to the development of scoliosis [26–31], which follows the Hueter-Volkmann principle [32]; growth is retarded by increased mechanical compression and accelerated by reduced loading. The morphological changes of the scoliosis are highly correlated with the asymmetric stress of intervertebral discs. Collectively, our data showed evidence that unilateral pedicle screw fixation can effectively reduce the pressure of the fixed side of the intervertebral disc and therefore be able to correct unbalanced stress.

When considering the average pressure, in the fixed group, the value of each segment was decreased compared to that in the unfixed group, suggesting that unilateral pedicle screw fixation can alleviate the overall stress of the intervertebral disc. We further calculated the percentage of the mean pressure of the fixed group to that of the unfixed group; results showed that the most contributable segments of the pedicle screw fixation system include the lower thoracic and thoracolumbar vertebrae.

Our study has several limitations. Firstly, porcine animal model with important differences to human beings (intradiscal pressure and anatomy) was used, because the availability of human cadaver material is very limited, particularly from the younger population. Therefore, the applicability of our strategy to human beings requires further study. Secondly, to compare the stress differences between the fixed side and the unfixed side, this study only focused on the unilateral pedicle screw fixation and the force of the spine was not fully revealed. Lastly, only some of the representative spinal segments were studied in this study, and the biomechanical data of the whole spine could not be fully reflected. Thus, further study regarding the biomechanical data of the whole spine and the difference between bilateral and unilateral pedicle screw fixation based on human beings is required in future.

## Conclusion

Unilateral pedicle screws can effectively reduce the pressure of the fixed lateral intervertebral disc. In addition, it can change the pressure distribution of the intervertebral disc and reduce the press. The pressure characteristics obtained from the present study may be helpful in understanding the effects of the pedicle screw fixation system on the treatment of scoliosis.
